# Cerebrospinal Fluid Biomarkers and Neuropsychological Abnormalities in Dementia: A Monocentric Study of Consecutive Patients

**DOI:** 10.3390/jcm14030710

**Published:** 2025-01-22

**Authors:** Martin Römer, Christian Lange-Asschenfeldt, Katharina Müller-Schmitz, Rüdiger J. Seitz

**Affiliations:** 1 Department of Neurology, Centre for Neurology and Neuropsychiatry, LVR-Klinikum Düsseldorf, Medical Faculty, Heinrich-Heine-University Düsseldorf, 40629 Düsseldorf, Germany; ma.roemer@web.de (M.R.); katharina.mueller-schmitz@lvr.de (K.M.-S.); 2 Department of Forensic Psychiatry, LVR-Klinikum Langenfeld, 40764 Langenfeld, Germany; c.lange.asschenfeldt@gmail.com

**Keywords:** Alzheimer’s dementia (AD), CSF biomarkers, neuropsychological tests, CERAD Plus, amyloid cascade hypothesis

## Abstract

**Background**: In search of indicators for dementia, this study investigated the association of cerebrospinal fluid (CSF) biomarkers and neuropsychological test results with disease stage in patients with early manifestations of dementia. **Methods**: In 190 consecutive patients with symptoms of dementia, the CSF parameters amyloid-β 1-42 (Aβ1-42), phosphorylated tau protein (pTau), total tau protein (tTau), neuron-specific enolase (NSE), protein S100B (S100B), and Aβ (1-42)/(1-40) ratio (Aβ ratio), as well as the results of the CERAD-Plus test battery supplemented by the Clock Drawing Test (CDT), were analysed. Patients were divided into two groups based on the median duration of reported symptom onset. **Results:** Most prominent in the early phase of the disease were the relationships between Aβ1-42 and neuropsychological memory subtests, which were absent in the later phase. Less pronounced relationships to memory function were detectable for Aβ ratio and pTau. **Conclusions:** The results substantiate the relevance of Aβ1-42 for memory deficits and support the amyloid cascade hypothesis for Alzheimer’s dementia (AD). Our data suggest other pathomechanisms for visuospatial impairments in AD.

## 1. Introduction

Dementia has a high socio-economic burden, which is of increasing importance in contemporary societies. While the number of people affected by dementia worldwide was around 57.3 million in 2019, 152.8 million people are expected to suffer from dementia by 2050 [1]. The main reason for this is demographic changes, with an increasing proportion of elderly people. In particular, in the currently young populations of African and Arab countries, the number of people with dementia is expected to be more than three times higher by 2050 than today [1]. Moreover, there will also be more people with dementia in the industrialised countries of North America (+102%) and Europe (+74%) [1]. The decrease of the number of younger people will slow down global population growth, resulting in a possible peak around 2050 and a shrinking world population afterwards [2]. As a result, there will be fewer young people to care for an increasingly number of patients with dementia in the future. Therefore, it is of great relevance to identify effective therapeutics against dementia, especially for Alzheimer’s disease (AD), as the most common form of dementia [3]. In the pathogenesis of this form of dementia, the so called amyloid cascade hypothesis, which places the peptide Aβ at the beginning of a pathological cascade, is widely recognised [4,5]. The antibodies Lecanemab (recently approved) and Donanemab, which are directed against Aβ1-42, achieved for the first time a clear clinical benefit in terms of delaying disease progression [6,7]. Starting treatment as early as possible, at a time when the neuronal damage in the brain is still limited, is considered important for the success of treatment approaches [8,9].

Accordingly, it is of the utmost importance to identify biomarkers that may contribute to pre-symptomatic identification of treatment candidates [10], especially since it is assumed that the underlying pathological processes in AD start many years before the clinical manifestation of the disease [11]. Our study, therefore, aimed to investigate the relationship between neurodegenerative markers in the CSF currently used to diagnose dementia on the one hand, and neuropsychological test results on the other hand. To estimate the power of these biomarkers for presymptomatic diagnosis we were particularly interested in exploring the influence of the disease stage on their associations.

For the diagnosis of AD, the criteria of the National Institute on Aging and the Alzheimer’s Association (NIA-AA) from 2011 require, among other items, an impairment of everyday activities. This criterion is considered to discriminate AD from MCI (mild cognitive impairment) [12]. Moreover, the NIA-AA criteria also require a slow onset of symptoms over months to years, with a clear cognitive deterioration over time and either typical memory impairment or atypical impairments, for example of speech. Furthermore, differential diagnoses have to be ruled out and biomarkers (CSF parameters, MRI, FDG-PET, and, recently, the Aβ1-42 ligand PET) should be included in the diagnostic procedure [12].

Whereas these guidelines are primarily based on clinical symptoms, the scientific focus has shifted recently to the in vivo biomarkers that are considered to be biological evidence for the presence of AD pathology regardless of the clinical manifestation [11]. In 2018, the NIA-AA proposed a diagnostic concept that is based on biomarkers of amyloid pathology (A), tau pathology (T), and general neurodestruction (N). AT (N) biomarkers focus primarily on the detection of Aβ plaques and tau fibrils, as in PET–CT or the corresponding changes in Aβ and pTau in CSF, while general neurodestruction (tTau, FDG-PET hypometabolism, and brain atrophy in cMRI) plays a subordinate role and only allows for an AD diagnosis in combination with the other two parameters [11].

However, due to substantial heterogeneity of neuropsychological deficits and variable results of CSF and imaging findings among patients with early and moderately advanced stages of dementia, clear assignment of a specific form of dementia is often not feasible. For example, in clinical practice, atypical or mixed forms of dementia are the rule rather than the exception and considerable inter-individual variations in characteristics occur even in identical diseases [13,14]. Moreover, neuropsychological tests often show poor sensitivity and specificity, particularly in the diagnostically and therapeutically important early phase [15]. Even in centres with great expertise and numerous years of clinical follow-up, the specificity and sensitivity of differentiating AD from other forms of dementia are only around 70% [16]. In addition, the differential diagnostic discriminatory power of CSF biomarkers within the group of neurodegenerative diseases, and in differentiation from vascular dementia (VaD), is seemingly also insufficient [17]. For example, typical AD CSF findings appear to influence treatment success for normal pressure hydrocephalus, which is actually regarded as a separate entity [18,19]. None the least, differentiation between AD and VaD is particularly difficult, because in the majority of elderly patients with advanced dementia, both vascular and neurodegenerative changes are detected at autopsy [20]. In fact, recent large studies suggest that mixed and multi-etiological dementia forms are the most common causes of cognitive impairment in old age [21,22]. Thus, clinical diagnosis in the early stages of dementia is still challenging and requires neuroimaging-differentiated neuropsychological testing and CSF studies of biomarkers of dementia. In consideration of the diagnostic shortcomings mentioned, this study on the possible relationship of neuropsychological deficits and biomarker abnormalities in CSF, deliberately did not take into account the clinical dementia diagnoses of the patients.

## 2. Methods

Patients: This retrospective study included patients who were treated consecutively in the Department of Neurology at the LVR Hospital Düsseldorf, Germany, between 2015 and 2022. The study was approved by the Ethics Committee of the Medical Faculty at Heinrich Heine University, Düsseldorf. The following inclusion criteria were applied: each patient was admitted because of symptoms of dementia; all patients completed neuropsychological testing with the CERAD Plus test battery; and each patient had a diagnostic lumbar puncture as part of the diagnostic process. Furthermore, the time span from the onset of symptoms (based on documented patient or caregiver information) was assessed. A total of 192 patients fulfilled these criteria and were included. The following exclusion criteria were applied: Patients for whom clinical symptoms of dementia had not been established were excluded from the study. These included patients with pseudodementia due to depression, metabolic disorders such as vitamin B12 deficiency, type 2 diabetes mellitus, and other conditions. Also, two patients were excluded as extreme outliers because their CSF concentrations of Aβ1-42 differed more than five interquartile ranges from the 75th percentile or more than five standard deviations from the mean. Based on their clinical data, the patients were subdivided into two groups using the median of reported disease duration, which was 12 months. Patients with a disease duration of 0–12 months were assigned to group A (n = 107, 56.3%), while patients with a disease duration of more than 12 months were assigned to group B (n = 83, 43.7%).

Investigations: Each patient underwent neuropsychological testing using the German version of the Consortium to Establish a Registry for AD (CERAD)-Plus test battery (www.memoryclinic.ch). The tests were carried out by qualified neuropsychologists. In some cases, test results were not complete, e.g., due to the weariness or inability of the patients, particularly in the case of *Trail Making Test B* (60% completeness). The test items of the CERAD-Plus included the following (percentage of completeness in our sample in brackets): *Word List Learning* (98.4%), *Word List Delayed Recall* (97.9%), *Word List Intrusions* (68.4%), *Word List Savings* (92.6%), *Word List Recognition* (97.4%), *Semantic Fluency* (97.4%), *Phonematic Fluency* (93.2%), *Trail Making Test A* (84.2%), *Trail Making Test B* (60%), *Boston Naming Test* (98.4%), *Mini Mental Status Test* (MMST) (97.9%), *Constructional Praxis* (93.7%), *Constructional Praxis Recall* (94.2%), and *Constructional Praxis Savings* (92.6%). In all these tests, z-standardized values were used. In most cases, the CERAD-Plus test battery was supplemented by a *Clock Drawing Test* (CDT) (94.7%).

The concentrations of Aβ1-42, pTau phosphorylated on threonine 181 (181P), six isoforms of tau protein as a marker for tTau, as well as NSE and S100B were analysed. Aβ1-42, pTau and tTau were determined using the ELISA-based tests INNOTEST^®^-AMYLOID (1-42), INNOTEST^®^ hTAU Ag, and INNOTEST^®^ PHOSPHO TAU (181P). NSE and S100B were measured using the fully automated chemiluminescence immunoassays LIASON^®^ S100 and LIAISON^®^ NSE from the Italian company DiaSorin. For the Aβ ratio, the ELISA test EUROIMMUN^®^ measured Aβ1-40, which was then compared with Aβ1-42. The reference values provided by the laboratory were used.

Statistics: Microsoft^®^ Excel for Windows, version 15.0, was used for primary data handling. Statistical analyses were carried out with IBM^®^ SPSS^®^ Statistics for Windows, version 27. Multiple linear regression analysis was performed by the backward elimination method. For group comparisons, we used unpaired t-tests. Correlations were calculated with the non-parametric Spearman’s rank correlation. In this study, α = 0.05 was selected and therefore *p* < 0.05 was considered significant for group comparisons and correlations.

As this study pursued an exploratory approach and did not test a specific hypothesis, we decided not to adjust the α level for multiple comparisons. Due to the multitude of CSF parameters and neuropsychological tests investigated, an evaluation with an adjusted α level was not expedient. We thus accepted the risk of committing type 1 errors (false positives) in order to keep the risk of type 2 errors (false negatives) acceptable.

## 3. Results

Demographic data: The 190 patients included 98 men (51.6%) and 92 women (48.4%). The mean age of the patients at the time of enrolment was 71 (+/− 9) years. The youngest patient was 52 years old and the oldest 92 years old. 49 patients (25.8%) were younger than 65 years at the time of inclusion and thus fulfilled the diagnostic criterion for pre-senile dementia. 113 patients (59.5%) were finally diagnosed with AD, 21 (11.1%) with FTD, and only five (2.6%) were considered to have VaD. 51 patients (26.8%) could not be assigned to any of these diagnosis groups. In these patients, the clinical diagnosis remained either vague (e.g., “cognitive deficits” or “dementia development”) or no clear distinction was possible. However, as described above, the analysis of the patients was independent of the clinical diagnoses.

We aimed to assess whether the stage of the illness had an influence on the CSF parameters and on the neuropsychological test results. Therefore, the patients were divided into two groups based on the median of the time span between symptom onset and enrolment into the study: patients in whom the symptoms had appeared no more than twelve months ago (Group A, 56.3%) and those who reported a cognitive impairment that had been present for more than one year (Group B, 43.7%). The patients in group A were slightly older (M = 72 ± 9 years) than those in group B (69 ± 8.0 years). In addition, the proportion of women in group A (55.1%) was significantly higher than in group B (39.8%).

**Figure 1:**
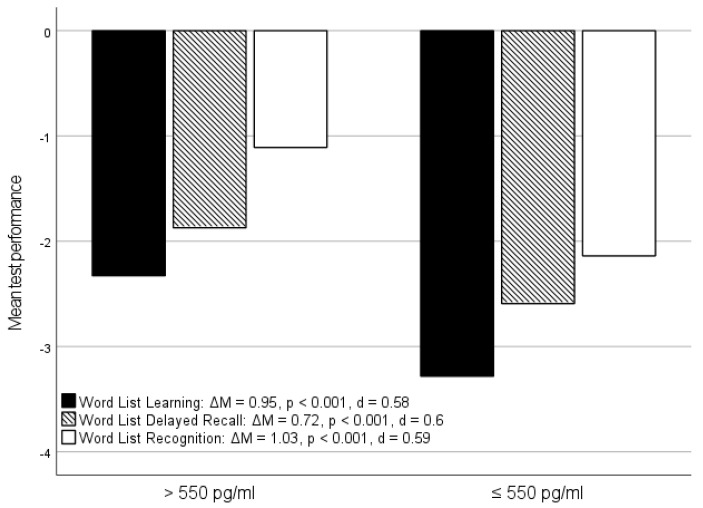
**Comparison of memory test performance in relation to Aβ1-42 status:** Mean values for the Word List Learning, Word List Delayed Recall, and Word List Recognition Tests in relation to Aβ1-42 status (>550 pg/mL = normal/≤550 pg/mL = pathological); ΔM = mean difference between the two groups; *p* = *p*-value; and d = effect size according to Cohen.

Associations between CSF parameters and neuropsychological testing: The CSF parameters showed that Aβ1-42 had the strongest relation with the results of the neuropsychological tests. The results of the neuropsychological tests and of the CSF parameters are detailed in Supplementary Tables S1 and S2. Reduced CSF concentrations of this biomarker were particularly associated with poor results in the memory subtests. Strong relationships were found with the *Word List Learning*, *Word List Delayed Recall,* and *Word List Recognition* tests (Figure 1): In the *Word List Learning* test, patients with normal Aβ1-42 values performed significantly better by an average of almost one SD than those with pathologically low Aβ1-42 concentrations (Figure 1). Furthermore, there was a significant, albeit weak, correlation between the results of this test and the Aβ1-42 concentration in the CSF (Table 1). In addition, Aβ1-42 was the most important predictor of the independent variable *Word List Learning*, calculated using multiple linear regression, ahead of Aβ quotient and pTau (R^2^ = 0.17, *p* < 0.001).

A similar result was obtained for the *Word List Delayed Recall* test. This verbal episodic memory test assesses the ability to retain newly learnt information in the long term. In AD, this part of the CERAD-Plus test battery is often the first to show abnormalities [23]. Here, there was also a significant difference between patients with normal and pathological Aβ1-42 values, although this was less pronounced than with *Word List Learning* at a comparable effect size (Figure 1). The results from *Word List Delayed Recall* and the Aβ1-42 concentration showed equally weak but significant correlations (Table 1). Again, the Aβ1-42 concentration was the most important predictor in the prediction model (beside Aβ quotient and pTau) with a moderate variance explanation (R^2^ = 0.15, *p* = 0.001).

The greatest mean difference was observed in *Word List Recognition*: Patients with normal Aβ1-42 concentrations performed more than one SD better than patients with low Aß1-42 concentrations (Figure 1). The correlation between the two parameters was again weak but robust (Table 1). In the multiple linear regression, Aβ1-42 was the only significant predictor for *Word List Recognition* (R^2^ = 0.07, *p* = 0.021).

**Figure 2:**
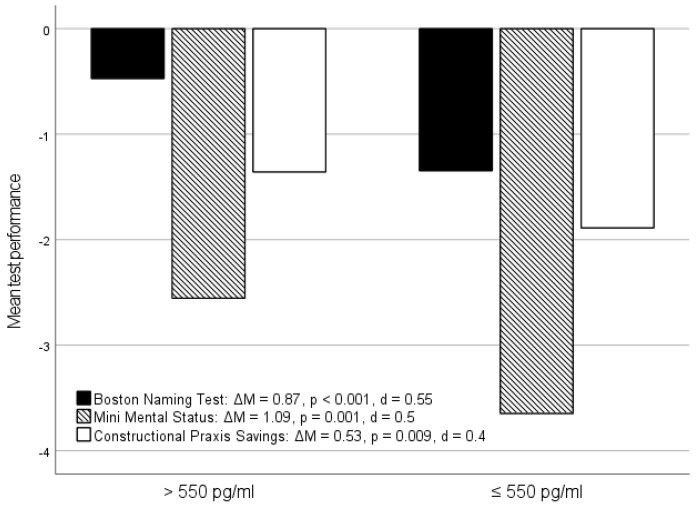
**Comparison of memory subtest performance in relation to the Aβ1-42 status:** Mean values for the Boston Naming Test, Mini Mental Status Test, and Constructional Praxis Savings in relation to Aβ1-42 status (>550 pg/mL = normative/≤550 pg/mL = pathological); ΔM = mean difference between the two groups; *p* = *p*-value; and d = effect size according to Cohen.

In addition to the specific memory subtests, other subtests of the CERAD-Plus battery also showed significant relationships with Aβ1-42, as follows. For the *Boston Naming Test*, a significant difference between normal and pathological Aβ1-42 concentrations was detectable (ΔM = 0.87, *p* < 0.001, d = 0.55, Figure 2). Both parameters showed a weak but significant correlation with each other (ρ = 0.203, *p* = 0.005, Table 1).

The largest mean difference between patients with pathological and normal Aβ1-42 concentrations was found for the MMST (Figure 2). This test also showed a significant, albeit weak, correlation with Aβ1-42 (Table 1). A small mean difference with only a small effect size was found in the *Constructional Praxis Savings* test (Figure 2). The weakest, but still significant correlation to Aβ1-42 was also detectable here (Table 1). For the *Constructional Praxis* and *CDT* tests, multiple linear regression did not reveal any relevant relationships with Aβ1-42 or other CSF parameters. However, we found significant differences and correlations between Aβ1-42 and *CDT* results, which were clearly less pronounced than the relationships between Aβ1-42 and the tests mentioned above. While the Aβ quotient has been considered in some studies as a possible and even superior alternative to Aβ1-42 [24,25], the correlations between this parameter and the neuropsychological test results in this study were far less pronounced than for Aβ1-42. The same applied to pTau, for which correlations and mean differences with the memory subtests could also be delineated, but these were weaker than for Aβ1-42 (Supplementary Table S3). For the biomarkers tTau, NSE and S100B, there were no relevant correlations or mean differences with the neuropsychological test results (Supplementary Tables S4–S6).

**Figure 3:**
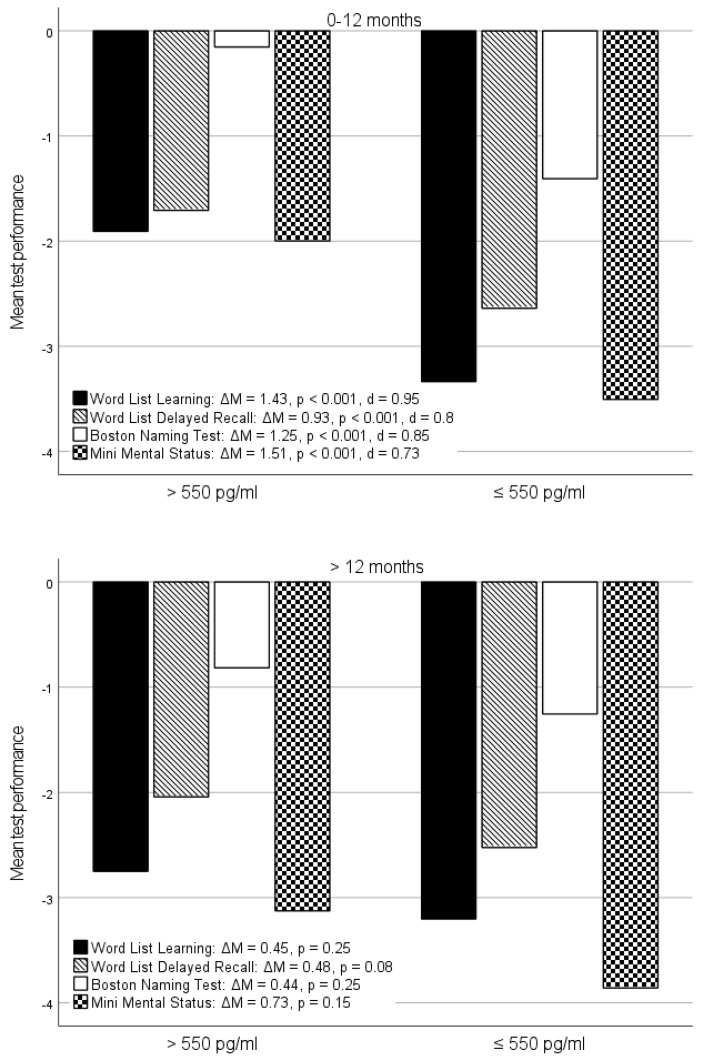
Neuropsychological tests in relation to Aβ1-42 status in patients with a longer duration of illness compared with those with a shorter duration: mean values for the Word List Learning, Word List Delayed Recall, Boston Naming Test, and Mini Mental Status Test in relation to the Aβ1-42 status (>550 pg/mL = normative/≤550 pg/mL = pathological) in Group A (0–12 months) and Group B (>12 months); ΔM = mean difference according to Aβ1-42 status; *p* = *p*-value; d = effect size according to Cohen.

Influence of the stage of illness: When the stage of illness was included in the analysis, significantly stronger relationships emerged, particularly for Aβ1-42, in the patients with a symptom onset of no more than twelve months (Group A) than in those who had already had dementia symptoms for more than a year (Group B). For the subtests *Word List Learning*, *Word List Delayed Recall*, *MMST*, *Boston Naming Test*, and *Constructional Praxis Savings* in Group A, larger mean differences were recorded between patients with normal and pathological CSF Aβ1-42 concentrations compared to the total sample. The effect sizes of the respective mean differences, which were mostly moderate in the total sample, were high for Group A in the *Word List Learning*, *Word List Recognition*, and *Boston Naming* Tests. For Group B, there were no differences between normal and pathological Aβ1-42 concentrations for any of the above-mentioned tests (Figure 3).

A similar result was found for the Spearman correlations between the neuropsychological test results and the CSF Aβ1-42 concentrations (Table 2). While these were weak in the overall sample for all subtests considered, there were moderate correlations in Group A for the *Word List Learning*, *Word List Delayed Recall*, *MMST*, and *Boston Naming Test*. However, no correlations were detected for these tests in Group B. The exception here was the *Word List Delayed Recall* test, for which a weak but significant correlation (ρ = 0.243) was detectable, although this was weaker than the moderate correlation in Group A (ρ = 0.337, Table 2).

## 4. Discussion

In this retrospective study of 190 patients who were investigated consecutively in this hospital because of symptoms of dementia, we found that impairments of memory as assessed with *Word List Learning*, *Word List Delayed Recall*, and *Word List Recognition* were strongly associated with CSF Aβ1-42 status. In addition to memory impairment as the cardinal symptom of AD and obligatory for its diagnosis [26], there were also strong associations between the *Boston Naming Test*, the *MMST*, and *Constructional Praxis Savings* and Aβ1-42. This is consistent with the broader range of cognitive deficits typically found in AD. While the *Boston Naming Test* assesses not only semantic memory but also visual perception and recognition as well as word finding [27,28], the *MMST* includes an array of cognitive items that probe short-term and episodic memory [29]. Moreover, the parameter *Constructional Praxis Savings*, which is defined as the ratio of the results of the *Constructional Praxis Recall* and *Constructional Praxis* subtests, is regarded as a measure of non-verbal retention [27]. Most importantly, however, our data confirm that memory impairments are prominently associated with reduced Aβ1-42 levels in CSF.

Furthermore, our results on the *Word List Delayed Recall* and the *Word List Recognition* tests point towards a possible distinction between memory encoding and recall deficits. While deficits in memory encoding are predominantly due to cortical and hippocampal changes, recall deficits are more likely to be due to subcortical pathology [27]. Therefore, the correlation of poor results in *Word List Delayed Recall* and good results in *Word List Recognition* points toward a deficit of recall, as learned and stored content can apparently only be recalled upon re-presentation of the words from the tests [27,30]. Thus, it was striking that the patients on average achieved much better results in the *Word List Recognition* test (M = −1.70) than in the *Word List Delayed Recall* subtest (M = −2.29), which suggests a recall disorder due to subcortical pathology. Accordingly, a substantial number of patients with subcortical, i.e., vascular dementia (VaD), would have been expected in our sample. However, only 2.6% of the patients had received a clinical diagnosis of VaD at discharge. It can, therefore, be assumed that the majority of our patients who were ultimately diagnosed with AD also exhibited subcortical damage with a recall disorder. This, in turn, can be considered to support the notion that the majority of senile dementias represent multi-etiological or mixed forms, in particular of AD and VaD [14,20,21,22].

Surprisingly, there were no or only clearly weaker relationships between the *CDT* and *Constructional Praxis* tests, which assess visuospatial and visuoconstructive skills, and CSF Aβ1-42 status. This is remarkable because the disabilities that result from dysfunction of the parietal cortex are considered to be typical symptoms of AD alongside memory deficits [31]. Nor were there relationships between the *Constructional Praxis Recall* test (which is based on *Constructional Praxis* and is intended to test non-verbal memory) and Aβ1-42 status in CSF. However, this test is known to be sensitive to disorders of visuospatial abilities. Patients who are already unable to draw the presented figures directly (*Constructional Praxis*) will also be unable to draw them correctly from memory (*Constructional Praxis Recall*), even if they have memorised the figures correctly [27,28]. To circumvent this problem, the CERAD-Plus test battery also includes the *Constructional Praxis Savings* parameter, which represents the ratio of the results of *Constructional Praxis Recall* and *Constructional Praxis*. Thereby, it eliminates the influence of visuoconstructive deficits representing non-verbal memory in isolation as a relative measure of non-verbal retention performance. In fact, we found an association of this test with Aβ1-42 status. These results suggest that visuospatial and visuoconstructive disorders may result from pathomechanisms other than the amyloid cascade, suggesting a multifactorial aetiology even for AD.

No relevant associations were found between any of the CERAD-Plus subtests and the CSF parameters S100B, NSE, and tTau in our patients with initial or clinically manifest dementia. In a previous study with a sample of prodromal and mild AD patients, we found strong associations between CSF S100B levels (indicative of neuroinflammation) and deficits in overall cognitive performance (CERAD-Plus total score) and in memory encoding (*Word List Learning*: delayed recall and overall performance) [32]. It can be conjectured that inflammatory processes might play a role in the pathogenesis of (very) early and mild, but not manifest AD/dementia, as in the present sample.

Our findings show that the three CSF parameters S100B, NSE, and tTau, do not allow for firm predictions of neuropsychological performance in dementia patients. This suggests that the cognitive impairments tested by the CERAD-Plus battery are possibly attributable to AD-specific cerebral changes that are not adequately represented by these unspecific destruction parameters, even in advanced stages (i.e., Group B in the present study). This is also supported by the fact that even for the *MMST*, which tests a whole array of cognitive domains and could therefore possibly show abnormalities in several functional systems in general neuronal damage, no relevant relationships with these unspecific destruction markers could be identified.

**Figure 4:**
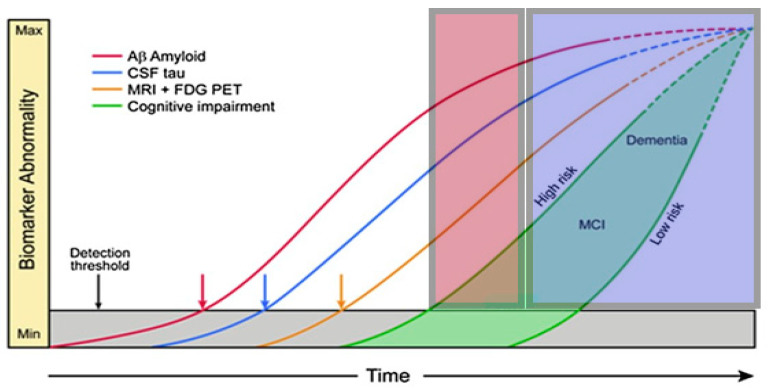
**Different disease duration in the amyloid cascade model:** Time course of biomarkers and cognitive impairment in Alzheimer’s dementia according to [37], modified to represent the patients with short disease duration (red box, Group A, 0–12 months) and longer disease duration (blue box, Group B, >12 months).

The stronger relationship of the memory subtests to Aβ1-42 in CSF in patients with less than one year of illness than in the patients with dementia symptoms for more than one year fits well with the amyloid cascade hypothesis. This hypothesis postulates that a number of interrelated neuropathological processes precede cognitive impairment in AD, sometimes for many years, and that they have already reached their peak levels while the clinical symptoms are still progressing [33]. Accordingly, it can be assumed that the correlations of the cognitive deficits with the neuropathological processes are more pronounced at the beginning of the clinical symptoms, i.e., at a time when both parameters are still in their dynamic phase, than at a later stage when the neuropathological processes are already more advanced. This hypothesis is illustrated in Figure 4: In the early phase of the clinically manifest disease marked by the red box, which corresponds to Group A in our study, the curves for Aβ1-42 (red) and the cognitive deficits (green) still run relatively parallel to each other. This changes with a longer disease duration depicted in the blue box, which corresponds to Group B in our study, when the Aβ1-42 concentration has reached its maximum while the cognitive decline continues to progress. This suggests that abnormalities of Aβ1-42 in CSF may allow for an early diagnosis of AD when a therapeutic intervention may still be able to prevent cognitive decline.

The lack of relevant differences between Group A and Group B for pTau may also be explained by this assumption. According to the amyloid cascade hypothesis, tau fibrils, and related pathology are possibly triggered by extracellular amyloid deposition and thus appear with a time delay, so that pTau concentrations reach their plateau later than Aβ1-42 [34,35]. Accordingly, similar differences would be expected for pTau as for Aβ1-42 if the separation into Groups A and B was made with regard to more advanced disease duration. On the contrary, however, a longitudinal study showed initially elevated concentrations of pTau in CSF, which then decreased over the course of the disease [36].

## 5. Limitations

The present study had an exploratory character and did not pursue specific hypotheses. With respect to the large number of calculations, we refrained from adjusting the α-level by α-correction to minimise the risk of type 2 errors occurring, i.e., the false rejection of the alternative hypothesis. On the other hand, this may have increased the risk of type 1 errors, so that some results may have been accepted incorrectly as significant. However, since the results were analysed in the overall clinical context, the risk of drawing fundamentally incorrect conclusions was probably low. Moreover, we deliberately included patients with initial or manifest memory deficits into this study, as we sought to explore the possible relationships of CSF biomarkers to the neuropsychological test results independently of clinical dementia diagnoses. We can, however, not exclude that this approach may weaken our conclusions regarding the amyloid cascade hypothesis to some extent. Finally, it should be mentioned that we had no control group. This was due to the fact that a lumbar puncture is potentially harmful and, thus, cannot be performed in healthy subjects or other patient groups unless for defined diagnostic purposes. Conversely, all patients presenting with symptoms of dementia in our hospital were subjected to lumbar punctures for diagnostic purposes.

## 6. Conclusions

The present study highlights the importance of the CSF parameter Aβ1-42 for the clinical diagnosis of dementia. Consistent with the currently widely recognized amyloid cascade hypothesis that puts intracellular degradation of the amyloid precursor protein (APP) into the centre of AD’s pathophysiology [37], impairments of neuropsychological memory subtests were related to lower CSF Aβ1-42 levels. While these results accord with today’s concept of AD, only markedly weaker relationships were found for visuospatial and visuoconstructive impairments, although they are regarded also as cardinal symptoms of AD. Thus, it may be speculated that the deterioration of visuospatial and visuoconstructive abilities may result from a different pathological mechanism.

## Figures and Tables

**Table 1:** Correlations between Aβ1-42 and neuropsychological test results in the total sample.

Aβ1-42	Word List Learning	Word List Delayed Recall	Word List Savings	Word List Recogn.	Phonematic Fluency	Boston Naming Test	Mini Mental Status	Constructional Praxis Savings	Clock Drawing Test
ρ	0.281	0.299	0.192	0.268	−0.070	0.203	0.223	0.164	−0.163
*p*	<0.001	<0.001	0.011	<0.001	0.351	0.005	0.002	0.030	0.028
n	187	186	176	185	177	187	186	176	180

Correlations between amyloid-β 1-42 (Aβ1-42) and the neuropsychological test results given by the Spearman correlation coefficient (ρ), *p*-value (*p*), and the number of patients (n).

**Table 2:** Correlations between amyloid-β 1-42 and neuropsychological tests according to disease stage.

Aβ1-42		Word List Learning	Word List Delayed Recall	Word List Savings	Word List Recogn.	Phonematic Fluency	Boston Naming Test	Mini Mental Status	Constructional Praxis Savings	Clock Drawing Test
0–12 months	ρ	0.387	0.337	0.186	0.251	0.021	0.400	0.352	0.163	−0.195
	*p*	<0.001	<0.001	0.067	0.011	0.834	<0.001	<0.001	0.107	0.052
	n	104	104	98	103	101	107	104	99	100
>12 months	ρ	0.164	0.243	0.151	0.259	−0.187	−0.022	0.098	0.153	−0.109
	*p*	0.138	0.028	0.188	0.019	0.106	0.846	0.382	0.184	0.337
	n	83	82	78	82	76	80	82	77	80

The correlations between amyloid-β 1-42 (Aβ1-42) and the neuropsychological test results in group A (0–12 months) and group B (>12 months). Spearman correlation coefficient (ρ), *p*-value (*p*), and number of patients (n).

## Data Availability

The datasheet with all data used for this study is available at Supplementary Materials.

## Supplementary Materials

The following tables are available online at www.mdpi.com/article/10.3390/jcm14030710/s1, Table S1: Descriptive data of neuropsychological tests, Table S2: Descriptive data of CSF parameters, Table S3: Correlations between phosphorylated tau protein and neuropsychological test results, Table S4: Correlations between total tau protein and neuropsychological test results, Table S5: Correlations between neuron-specific enolase and neuropsychological test results, Table S6: Correlations between protein S100B and neuropsychological test results. Table S7: The datasheet.

## References

[1] Nichols E., Chalek J., Steinmetz J.D., Stein E.V. (2022). Estimation of the global prevalence of dementia in 2019 and forecasted prevalence in 2050: An analysis for the Global Burden of Disease Study 2019. Lancet Public Health.

[2] United Nations (2019). World Population Prospects The 2019 Revision—Volume I: Comprehensive Tables.

[3] Förstl H., Kurz A. (1999). Clinical features of Alzheimer’s disease. Eur. Arch. Psychiatry Clin. Neurosci..

[4] Hardy J., Higgins G.A. (1992). Alzheimer’s Disease: The Amyloid Cascade Hypothesis. Science.

[5] Selkoe D.J., Hardy J. (2016). The amyloid hypothesis of Alzheimer’s disease at 25 years. EMBO Mol. Med..

[6] Van Dyck C.H., Swanson C.J., Aisen P., Bateman R.J., Chen C., Gee M., Kanekiyo M., Li D., Reyderman L., Cohen S. (2023). Lecanemab in Early Alzheimer’s Disease. N. Engl. J. Med..

[7] Sims J.R., Zimmer J.A., Evans C.D., Lu M., Ardayfio P., Sparks J., Wessels A.M., Shcherbinin S., Wang H., Monkul Nery E.S. (2023). Donanemab in Early Symptomatic Alzheimer Disease: The TRAILBLAZER-ALZ 2 Randomized Clinical Trial. JAMA.

[8] Khan S., Barve K.H., Kumar M.S. (2020). Recent Advancements in Pathogenesis, Diagnostics and Treatment of Alzheimer’s Disease. Curr. Neuropharmacol..

[9] Yiannopoulou K.G., Papageorgiou S.G. (2020). Current and Future Treatments in Alzheimer Disease: An Update. J. Cent. Nerv. Syst. Dis..

[10] Bjerke M., Engelborghs S. (2017). Cerebrospinal Fluid Biomarkers for Early and Differential Alzheimer’s Disease Diagnosis. J. Alzheimers’ Dis..

[11] Jack C.R., Bennett D.A., Blennow K., Carrillo M.C., Dunn B., Haeberlein S.B., Holtzman D.M., Jagust W., Jessen F., Karlawish J. (2018). NIA-AA Research Framework: Toward a biological definition of Alzheimer’s disease. Alzheimer’s Dement. J. Alzheimer’s Assoc..

[12] McKhann G.M., Knopman D.S., Chertkow H., Hyman B.T., Jack C.R., Kawas C.H., Klunk W.E., Koroshetz W.J., Manly J.J., Mayeux R. (2011). The diagnosis of dementia due to Alzheimer’s disease: Recommendations from the National Institute on Aging-Alzheimer’s Association workgroups on diagnostic guidelines for Alzheimer’s disease. Alzheimer’s Dement. J. Alzheimer’s Assoc..

[13] Custodio N., Montesinos R., Lira D., Herrera-Pérez E., Bardales Y., Valeriano-Lorenzo L. (2017). Mixed dementia: A review of the evidence. Dement. Neuropsychol..

[14] Kovacs G.G., Milenkovic I., Wöhrer A., Höftberger R., Gelpi E., Haberler C., Hönigschnabl S., Reiner-Concin A., Heinzl H., Jungwirth S. (2013). Non-Alzheimer neurodegenerative pathologies and their combinations are more frequent than commonly believed in the elderly brain: A community-based autopsy series. Acta Neuropathol..

[15] Boller F., Barba G.D. (2001). Neuropsychological tests in Alzheimer’s disease. Aging Clin. Exp. Res..

[16] Beach T.G., Monsell S.E., Phillips L.E., Kukull W. (2012). Accuracy of the clinical diagnosis of Alzheimer disease at National Institute on Aging Alzheimer Disease Centers, 2005–2010. J. Neuropathol. Exp. Neurol..

[17] Nägga K., Gottfries J., Blennow K., Marcusson J. (2002). Cerebrospinal fluid phospho-tau, total tau and beta-amyloid(1-42) in the differentiation between Alzheimer’s disease and vascular dementia. Dement. Geriatr. Cogn. Disord..

[18] Müller-Schmitz K., Krasavina-Loka N., Yardimci T., Lipka T., Kolman A.G.J., Robbers S., Menge T., Kujovic M., Seitz R.J. (2020). Normal Pressure Hydrocephalus Associated with Alzheimer’s Disease. Ann. Neurol..

[19] Klemke L.L., Müller-Schmitz K., Kolman A., Seitz R.J. (2023). Evolution of neurodegeneration in patients with normal pressure hydrocephalus: A monocentric follow up study. Neurol. Res. Pract..

[20] Power M., Mormino E., Soldan A., James B., Yu L., Armstrong N., Bangen K., Delano-Wood L., Lamar M., Lim Y.Y. (2018). Combined neuropathological pathways account for age-related risk of dementia: Multiple Pathologies and Age-Related Dementia Risk. Ann. Neurol..

[21] Kapasi A., DeCarli C., Schneider J.A. (2017). Impact of multiple pathologies on the threshold for clinically overt dementia. Acta Neuropathol..

[22] Schneider J.A., Arvanitakis Z., Bang W., Bennett D.A. (2007). Mixed brain pathologies account for most dementia cases in community-dwelling older persons. Neurology.

[23] Gallagher D., Mhaolain A.N., Coen R., Walsh C., Kilroy D., Belinski K., Bruce I., Coakley D., Walsh J.B., Cunningham C. (2010). Detecting prodromal Alzheimer’s disease in mild cognitive impairment: Utility of the CAMCOG and other neuropsychological predictors. Int. J. Geriatr. Psychiatry.

[24] Blennow K., Zetterberg H. (2018). Biomarkers for Alzheimer’s disease: Current status and prospects for the future. J. Intern. Med..

[25] Spies P.E., Slats D., Sjögren J.M.C., Kremer B.P.H., Verhey F.R.J., Rikkert M.G.M.O., Verbeek M.M. (2010). The cerebrospinal fluid amyloid beta42/40 ratio in the differentiation of Alzheimer’s disease from non-Alzheimer’s dementia. Curr. Alzheimer Res..

[26] (2022). Neurocognitive Disorders. Diagnostic and Statistical Manual of Mental Disorders.

[27] Aebi C. (2002). Validierung der Neuropsychologischen Testbatterie CERAD-NP: Eine Multi-Center Studie. Doctoral Dissertation.

[28] Rabitsch S. (2014). Die CERAD-Testbatterie. Psychopraxis Neuropraxis.

[29] Folstein M.F., Folstein S.E., McHugh P.R. (1975). Mini-mental state: A practical method for grading the cognitive state of patients for the clinician. J. Psychiatr. Res..

[30] Cummings J.L., Benson D.F. (1984). Subcortical dementia. Review of an emerging concept. Arch. Neurol..

[31] Quental N.B.M., Brucki S.M.D., Bueno O.F.A. (2009). Visuospatial function in early Alzheimer’s disease: Preliminary study. Dement. Neuropsychol..

[32] Christl J., Verhülsdonk S., Pessanha F., Menge T., Seitz R., Kujovic M., Höft B., Supprian T., Lange-Asschenfeldt C. (2019). Association of Cerebrospinal Fluid S100B Protein with Core Biomarkers and Cognitive Deficits in Prodromal and Mild Alzheimer’s Disease. J. Alzheimer’s Dis..

[33] Jack C.R., Holtzman D.M. (2013). Biomarker modeling of Alzheimer’s disease. Neuron.

[34] Blennow K., Dubois B., Fagan A.M., Lewczuk P., de Leon M.J., Hampel H. (2015). Clinical utility of cerebrospinal fluid biomarkers in the diagnosis of early Alzheimer’s disease. Alzheimer’s Dement. J. Alzheimer’s Assoc..

[35] Hardy J., Selkoe D.J. (2002). The amyloid hypothesis of Alzheimer’s disease: Progress and problems on the road to therapeutics. Science.

[36] Hampel H., Buerger K., Kohnken R., Teipel S.J., Zinkowski R., Moeller H.J., Rapoport S.I., Davies P. (2001). Tracking of Alzheimer’s disease progression with cerebrospinal fluid tau protein phosphorylated at threonine 231. Ann. Neurol..

[37] Thinakaran G., Koo E.H. (2008). Amyloid precursor protein trafficking, processing, and function. J. Biol. Chem..

