# Supplementary material for: Cerebrospinal Fluid Biomarkers and Neuropsychological Abnormalities in Dementia: A Monocentric Study of Consecutive Patients
Source: J Clin Med. 2025 Jan 22;14(3):710. doi: 10.3390/jcm14030710 (PMC11818742; doi:10.3390/jcm14030710)
Supplement: Supplementary file 1 [file jcm-14-00710-s001.zip › jcm-3362275-supplementary material.pdf]

Supplementary material

Table S1. Descripitive statistics of neuropsychological tests.

	n	M	SD	Minimum	Maximum
Word List Learning	187	-2,87	1,71	-7,52	1,55
Word List Delayed Recall	186	-2,29	1,25	-4,74	0,83
Word List Intrusions	130	-0,44	1,24	-3,33	2,83
Word List Savings	176	-1,76	2,02	-6,03	5,93
Word List Recognition	185	-1,70	1,81	-5,62	2,74
Semantic Fluency	185	-1,90	1,31	-5,39	1,88
Phonematic Fluency	177	-0,97	1,32	-4,69	2,00
Trail Making Test A	160	-1,43	1,49	-4,24	2,58
Trail Making Test B	114	-1,36	1,44	-6,75	2,41
Clock Drawing Test	180	2,71	1,37	1,0	6,0
Boston Naming Test	187	-0,98	1,64	-5,51	1,89
Mini Mental Status	186	-3,18	2,25	-9,72	1,45
Constructional Praxis	178	-0,88	1,69	-5,89	1,64
Constructional Praxis Recall	179	-2,09	1,62	-6,64	1,76
Constructional Praxis Savings	176	-1,66	1,34	-4,35	1,52

Number (n), mean (M) and standard deviation (SD) as well as the lowest (Minimum) highest score after z-standardization (Maximum) of the results of neuropsychological tests.

Table S2. Descriptive statistics of CSF-parameters.

	n	M	SD	Minimum	Maximum
Aβ1-42 [pg/ml]	190	576,06	314,44	80,0	1643,0
Aβ ratio	87	1,02	0,52	0,41	2,26
tTau [pg/ml]	189	632,60	363,85	133,0	2290,0
pTau [pg/ml]	188	81,34	49,21	10,8	259,0
NSE [μg/l]	188	19,27	7,80	4,4	52,7
S-100B [pg/ml]	177	3579,03	1306,63	652	11230

Number (n), mean (M) and standard deviation (SD) as well as the lowest (Minimum) and the highest score (Maximum) of CSF parameters. Aβ1-42 = Amyloid-β 1-42, Aβ ratio = Amyloid-β-ratio, tTau = total tau protein, pTau = phosphorylated tau protein, NSE = neuron specific enolase, S100B = protein S100B.

Table S3. Correlations between phosphorylated tau protein and neuropsychological test results.

**pTau**	Word List Learning	Word List Delayed Recall	Word List Savings	Word List Recognition	Phonematic Fluency	Boston Naming Test	Mini Mental Status	Constructional Praxis Savings	Clock Drawing test
ρ	-0,242	-0,152	-0,021	-0,175	0,141	-0,084	-0,136	-0,191	0,143
p	0,001	0,040	0,780	0,018	0,062	0,258	0,065	0,012	0,058
n	185	184	174	183	175	185	184	174	178

Correlations between phosphorylated tau protein (pTau) and the neuropsychological test results are shown with Spearman's coefficient of correlation (ϱ), p and number of included patients (n).

Table S4. Correlations between total tau protein and neuropsychological test results.

**tTau**	Word List Learning	Word List Delayed Recall	Word List Savings	Word List Recognition	Phonematic Fluency	Boston Naming Test	Mini Mental Status	Constructional Praxis Savings	Clock Drawing test
ρ	-0,177	-0,067	0,069	-0,103	0,072	-0,042	-0,146	-0,105	0,115
p	0,016	0,365	0,363	0,163	0,339	0,565	0,047	0,167	0,125
n	186	185	176	184	176	186	185	175	179

Correlations between total tau protein (tTau) and the neuropsychological test results are shown with Spearman's coefficient of correlation (ϱ), p and number of included patients (n).

Table S5. Correlations between neuron specific enolase and neuropsychological test results.

NSE	Word List Learning	Word List Delayed Recall	Word List Savings	Word List Recognition	Phonematic Fluency	Boston Naming Test	Mini Mental Status	Constructional Praxis Savings	Clock Drawing test
ρ	-0,051	0,044	0,133	-0,018	0,102	-0,027	0,004	-0,011	-0,053
p	0,493	0,556	0,080	0,812	0,181	0,711	0,957	0,884	0,478
n	185	184	174	183	175	185	184	175	179

Correlations between neuron specific enolase (NSE) and the neuropsychological test results are shown with Spearman's coefficient of correlation (ϱ), p and number of included patients (n).

Table S6. Correlations between protein S100 and neuropsychological test results.

S100	Word List Learning	Word List Delayed Recall	Word List Savings	Word List Recognition	Phonematic Fluency	Boston Naming Test	Mini Mental Status	Construction al Praxis Savings	Clock Drawing test
ρ	0,060	0,000	-0,026	0,065	0,120	0,013	0,105	0,062	-0,115
p	0,429	0,997	0,735	0,394	0,123	0,869	0,169	0,426	0,135
n	174	173	166	172	166	174	175	167	169

Correlations between protein S100 and the neuropsychological test results are shown with Spearman's coefficient of correlation (ϱ), p and number of included patients (n).
